# Epidemiological patterns of infectious and zoonotic diseases among dromedary camels in Saudi Arabia: a systematic review and meta-analysis of 79,154 camel samples

**DOI:** 10.1016/j.onehlt.2026.101618

**Published:** 2026-10-13

**Authors:** Ahmad T. Sabri Sabbah, Mai Abdel Haleem A. Abusalah, Alqassem Hamdallah Abuarqoub, Anwar Noor Althubyani, Fatmah Abdualrzg Albalwi, Naveed Ahmed

**Affiliations:** a Department of Assistance Medical Sciences, Applied College, University of Tabuk, 71491 Tabuk, Saudi Arabia; b Department of Medical Laboratory Sciences, Faculty of Allied Medical Sciences, Al-Ahliyya Amman University, Amman, Jordan; c Research Unit, Al-Wafi Group for Marketing and Int'l Trade Co. Ltd, Amman, Jordan

**Keywords:** Zoonotic diseases, Animal infections, One health, meta-analysis, Systematic review, Saudi Arabia

## Abstract

Dromedary camels are resilient, comparatively tolerant of several diseases, and can survive with fewer resources than other domestic farm animals. This systematic review and meta-analysis aimed to determine the pooled prevalence of infectious agents among dromedary camels in Saudi Arabia. After screening 804 records, we included 87 studies for qualitative and quantitative synthesis. The overall pooled prevalence of infection was 24.0% (95% CI: 0.18–0.32), with considerable heterogeneity (*I*^2^ = 99.4%, *p* < 0.001). Pathogen-specific pooled prevalences were 27.0% for parasites, 20.0% for viruses, 18.0% for bacteria, and 14.0% for fungi. Geographically, most studies originated from the Central region, and temporally, the majority were conducted between 2011 and 2020. Quality assessment using the JBI tool showed consistently high quality across all studies. However, Egger's test revealed publication bias (*p* = 0.006). These findings reinforce a large and heterogeneous level of infection in camels in Saudi Arabia, with parasitic infections showing the highest prevalence, suggesting their dominant role in shaping camel health dynamics. Hence, underscoring the need for integrated One Health surveillance and targeted control strategies to mitigate potential public health risks. The wide confidence intervals and high heterogeneity further reflect variability across studies, likely due to differences in geographic regions, diagnostic methods, and study designs.

## 1 Introduction

In Saudi Arabia, the dromedary camel (*Camelus dromedarius*) shows a distinct set of qualities in their genetics due to their adaptation to different climatic regions such as deserts, mountains, and coasts [1]. They hold substantial cultural, economic, and food security importance, as they contribute to meat and milk production, traditional livelihoods, and the national agricultural economy [2]. Camel farming systems range from extensive pastoral practices to increasingly intensive production systems, facilitating close human-animal interactions. These dynamics not only support economic sustainability but also create pathways for transmitting infectious diseases, including zoonotic diseases [3]. Understanding the epidemiology of infectious diseases in camel populations is therefore essential to safeguard animal health, ensure food safety, and protect public health within the Kingdom.

The ongoing incidence of zoonotic diseases, particularly Middle Eastern respiratory syndrome coronavirus (MERS-CoV), together with many other diseases like trypanosomiasis, brucellosis, and *Clostridium perfringens*, highlights the importance of strengthening routine surveillance. In the Middle East, promoting investment in diagnostic facilities, training programs, and strategies is important to address this issue at the camel-human interface [4]. Two zoonoses shown to have a major impact on farmed animals such as camels are Brucellosis and Q fever. They are caused by Gram-negative bacteria *Brucella* spp. and *Coxiella burnetii*, respectively, which can trigger abortions in bovines, small ruminants, and camels [5].

Camel immunology is also receiving significant attention. Many recent reviews have covered improvements in camel immunoglobulins. The camel defense system comprises a complex network of cellular and non-cellular components that support powerful immune responses against pathogens. The cellular compartment of the camel immune system has received less attention, while more effort has focused on camel immunoglobulins [6]. The health determines the prosperity of a livestock keeper. Camel keepers must understand this so they can identify signs as early as possible. Early Detection of diseases leads to Improved treatment outcomes. The health is closely linked to human health. Camel keepers need awareness of zoonotic diseases to protect themselves from pathogens and produce safe food for the nation [7].

Zoonotic pathogens affect people who live close to livestock in Saudi Arabia and other poor communities worldwide [8]. Zoonotic diseases that affect humans and are transmitted from animals are a major global challenge. Recent breakthroughs in molecular diagnostic methods have documented several pathogens in the literature [3]. Although numerous studies have investigated infectious diseases in camel populations within Saudi Arabia, these findings remain fragmented and pathogen-specific, limiting their broader applicability. To date, to the best of our knowledge, no comprehensive, quantitative synthesis integrates available evidence to estimate the overall burden and distribution of infectious diseases in camels at a national level.

From a public health perspective, dromedary camels represent a critical zoonotic interface in Saudi Arabia due to their close contact with humans, cultural significance, food value, and role in regional and transboundary trade [3], [4], [5]. Estimating the prevalence of infection in camel populations is thus critical not only in veterinary disease control measures but also in risk assessment for zoonoses, occupational health protection, food safety regulations, and readiness in emerging infectious diseases under a One Health platform. Hence, this systematic review and meta-analysis was conducted to (1) estimate the pooled prevalence of bacterial, viral, parasitic, and fungal infections in dromedary camels in Saudi Arabia; (2) explore sources of heterogeneity through subgroup and meta-regression analyses; and (3) discuss the findings within a One Health framework to inform public health and veterinary policy.

## 2 Material and methods

### 2.1 Protocols and registration

The protocol for this current research work was pre-registered with the International Prospective Register of Systematic Reviews PROSPERO, which allocated a registration number CRD420251053310. As part of planning this research work and before registering with PROSPERO, a systematic search with pre-defined keywords and eligibility criteria was undertaken in order to gauge not only the feasibility of conducting this research work but also to examine if a sufficient number of studies are available for this research work. Such a procedure for conducting this research work in a systematic way is part of following guidelines set by a systematic protocol, which is undertaken before conducting a search in various databases. The systematic review in this research work has followed the updated guidelines of PRISMA.

### 2.2 Literature search

A literature search with a focus on locating research articles that have recorded camel infections, including parasitic, bacterial, fungal, and viral infections in camels in Saudi Arabia, was conducted using a combination of online medical literature databases such as Scopus, PubMed, Web of Science, ScienceDirect, Google Scholar, and Cochrane Library. A strategy of using a combination of both MeSH keywords and non-standard keywords in an attempt to search for literature on the topic was used. Boolean searches using AND/OR were used to join search keywords whilst using filters to direct searching toward relevant journals. A literature search by screening the references in relevant articles was undertaken in order to search for relevant literature. The search results were all exported to a citation management tool, EndNote X20, to allow removal of duplicate records. A detailed search strategy has been provided in **Supplementary File 1**.

### 2.3 Inclusion and exclusion criteria

This systematic review focused on original studies illustrating the presence, prevalence, or identification of bacterial, viral, or fungal infection in dromedary camels in Saudi Arabia. Eligible studies included cross-sectional, cohort, case-control, case series, and surveillance studies reporting prevalence of infectious diseases in camels. Only studies published in English were included. We applied no restrictions on publication year and considered all studies available up to August 2025. Experimental or intervention studies (e.g., vaccine trials or therapeutic studies) were considered if they provided baseline data on infection status or prevalence before intervention. Studies primarily designed to evaluate diagnostic test performance without reporting prevalence estimates in camel populations were excluded. However, we included studies reporting prevalence of infectious diseases among camels, regardless of the diagnostic method used (e.g., serology, molecular assays, culture-based techniques). We also excluded published reports, conference proceedings, government or institutional surveillance bulletins, reviews, editorials, letters, and studies lacking primary data.

### 2.4 Data extraction

Data from the included studies were independently extracted by two reviewers (and verified by a third reviewer where applicable) using standardized Microsoft Excel sheets. Reviewers resolved discrepancies through discussion and consensus; if disagreement persisted, a third reviewer provided final adjudication. Extracted data included study name (author name and year of publication), study period, study design, method of diagnosis for infectious diseases, total number of studied cases, number of positive cases, pathogen type, and infection type. We categorized pathogens into viral, bacterial, and parasitic groups to enable a structured, system-level evaluation of infectious disease burden within a One Health context, while recognizing inherent biological heterogeneity; therefore, we incorporated subgroup- and pathogen-specific interpretations to ensure epidemiological relevance.

### 2.5 Data analysis

A combination of quantitative (meta-analysis) and narrative (descriptive) synthesis was employed. The primary approach involved aggregation of individual study-level data. No individual participant data were collected due to the nature of the included studies (animal population-level data). Statistical analyses were performed using multiple software tools, including R (meta/metafor packages), OpenMeta[Analyst], and Comprehensive Meta-Analysis (CMA), to ensure analytical robustness and cross-validation of results. R was primarily used for advanced statistical modeling and generation of forest plots, while OpenMeta[Analyst] and CMA were used for pooled prevalence estimation and verification of meta-analytic outputs.

We applied a random-effects model to estimate pooled prevalence, accounting for within- and between-study variability, as substantial heterogeneity across studies was anticipated due to variations in geographic regions, study designs, camel populations, pathogens investigated, and diagnostic methods. We presented the pooled estimate as a summary measure of overall disease burden and interpreted it alongside subgroup and meta-regression analyses to account for substantial heterogeneity.

We calculated pooled prevalence estimates for parasitic, bacterial, viral, and fungal infections. Where studies were too heterogeneous in design, outcomes, or statistical reporting to permit meta-analysis, a narrative synthesis was provided. This included structured descriptions of study characteristics, findings, and trends over time or by region. The decision to use quantitative vs. narrative synthesis was made per outcome domain based on the consistency of reported measures, availability of standard errors or confidence intervals (CI), and number of studies available.

The methods used for meta-analysis were the inverse-variance method, restricted maximum-likelihood estimator for tau^2, Q-Profile method for CI of tau^2 and tau, calculation of *I*^2 based on Q, Hartung-Knapp adjustment for random effects model, prediction interval based on t-distribution, Logit transformation, and Clopper-Pearson CI for individual studies.

A subgroup analysis was conducted to explore possible sources of heterogeneity and to identify differential infection patterns across key study characteristics. The planned subgroup analysis was based on the type of pathogen (bacterial vs. viral vs. fungal), geographical region within Saudi Arabia (e.g., central, western, eastern provinces), and the study period. Results from subgroup analyses were reported narratively and, where possible, stratified pooled prevalence estimates were generated.

### 2.6 Assessment of study bias and quality

The quality of the studies undertaken in this systematic review was assessed using the Joanna Briggs Institute (JBI) Critical Appraisal Checklist for prevalence studies. Two independent reviewers evaluated all studies based on a variety of domains such as representativeness of samples, case definition, methods of data collection, and results of outcomes. Consensus was achieved where there were any differences in evaluation. Bias in studies was measured in terms of low, moderate, or high.

## 3 Results

The search undertaken in this systematic review began in a variety of literature databases, including PubMed, Google Scholar, Scopus, Web of Science, ScienceDirect, and the Cochrane Library. A total of 804 articles were obtained in this search. Before screening, a total of 250 duplicates were identified. Of this total of 554 articles after eliminating duplicates, 383 articles were excluded based on title and abstract screening. Among the 161 articles considered for full-text eligibility, nine articles were not accessible, leaving a total of 152 articles for final evaluation. Of these articles, a total of 65 articles were considered not suitable for this systematic review & meta-analysis. A total of 65 were excluded based on predefined criteria, resulting in 87 studies included in the final analysis **(**Fig. 1**)**.

**Fig. 1:**
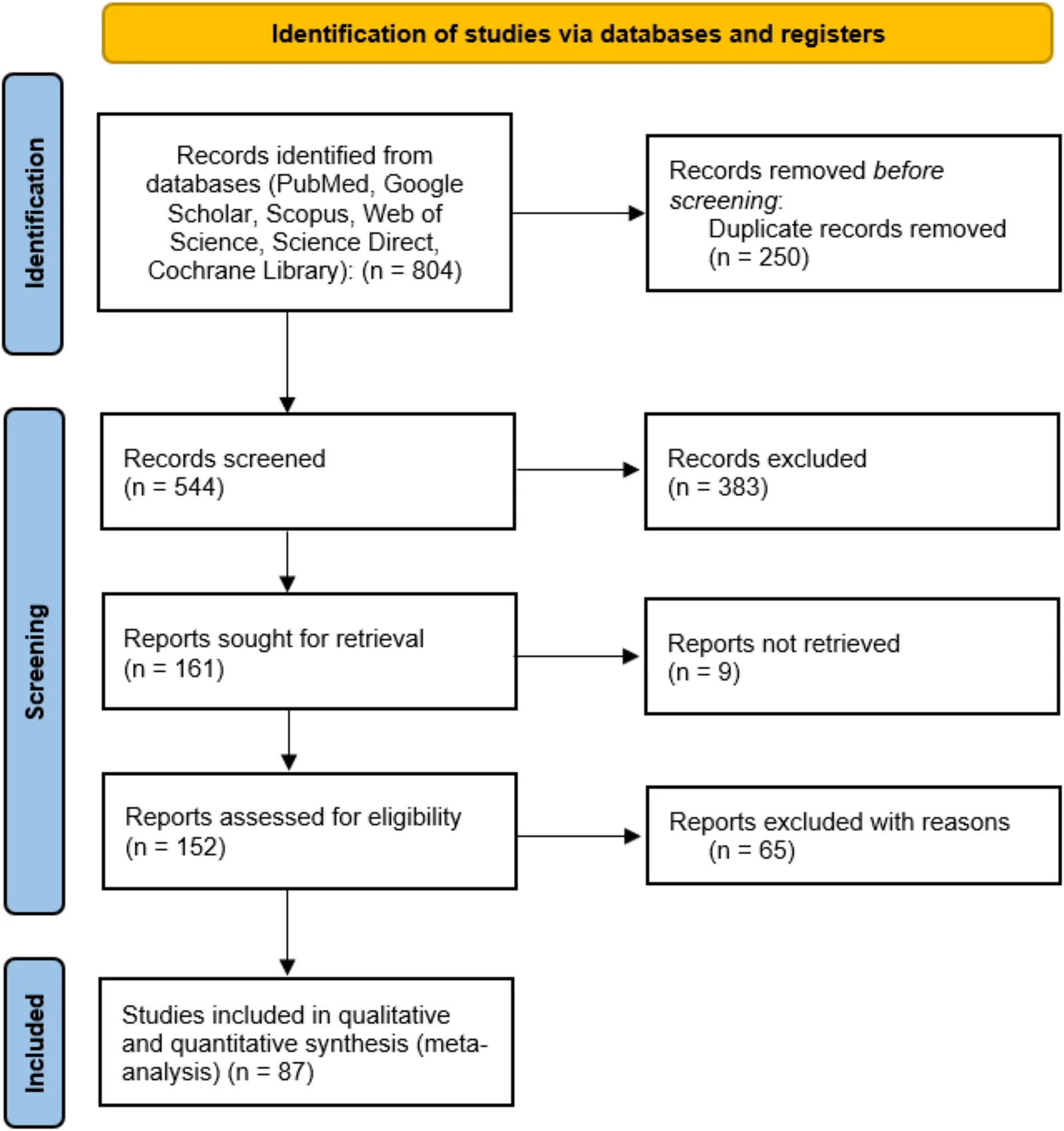
PRISMA flowchart for the reports screened and retrieved for the current study.

As illustrated in Fig. 2, the pooled prevalence of a given infection in camels in Saudi Arabia was estimated for the period from 1985 to 2025. The variability in prevalence estimation presented in these studies was quite large, with a range from 0.2% [9] to a high of 95.5% [10], indicating substantial heterogeneity in findings across different locations, times, and study populations. The pooled prevalence based on all included studies with a total of 79,154 camels is 24.4% (95% CI: 18.4%–31.5%). A very high value of the *I*^*2*^ statistic of 99.4% (*P* < 0.001) reveals a high degree of statistical heterogeneity among the studies, which means that the differences in the prevalence measures among studies are not due to chance but may be accounted for by differences in methods of diagnosis, geographical regions, and research studies being conducted. The key features of the studies used in this systematic analysis are given in Table 1.

**Fig. 2:**
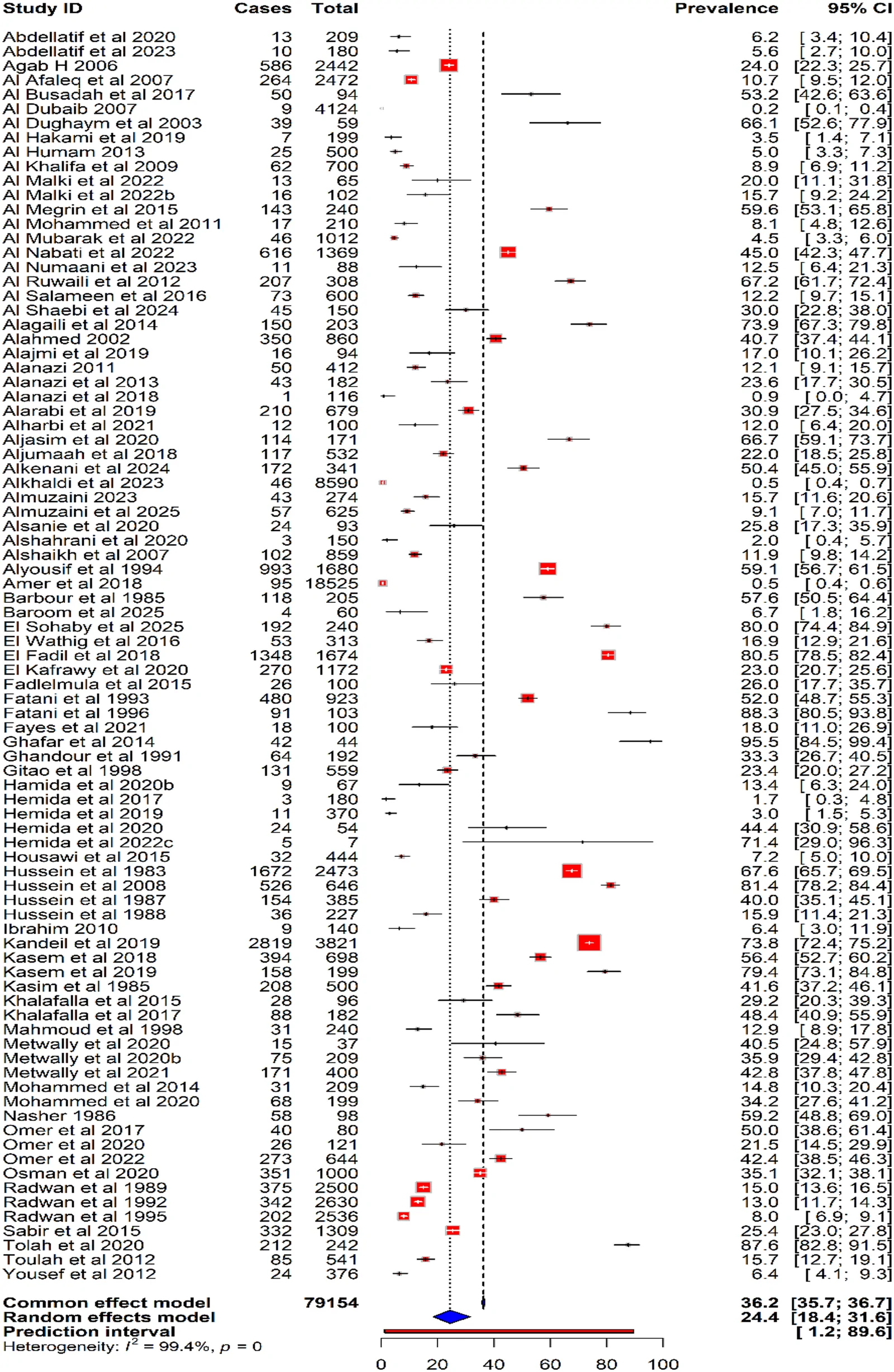
Forest plot of the pooled prevalence of infection rates among camels in Saudi Arabia.

**Table 1:** Major characteristics of reports retrieved for the current study.

Study reference	Study duration	City/Region	Diagnosis method	Total studied cases	Prevalence	Organism name
[11]	2011–2020	Northern region	RBPT, PCR, Sequencing	209	13	*Brucella* spp.
[12]	2021–2025	Northern region	RBPT, PCR, Sequencing	180	10	*Brucella* spp.
[13]	2001–2010	Central region	NA	2442	586	PPV, Trypanosomes, other undefined organisms
[14]	NA	Eastern region	Immunoassay	2472	264	Rinderpest, Bovine viral diarrhoea, Bovine rhinotracheitis, Rift Valley fever
[15]	2011–2020	Multiple regions	PCR	94	50	*Mycoplasma, Leptospira, Brucella*, BVDV
[9]	2001–2010	Central region	NA	4124	9	Rabies virus
[16]	2001–2010	Eastern region	Bacterial culture	59	39	*Proteus mirabilis, Staphylococcus aureus, Arcanobacterium pyogenes, Corynebacterium pseudotuberculosis, Pseudomonas aeruginosa and Streptococcus pyogenes, Candida krusei, Cryptococcus laurentii, Aspergillus penicillioides*
[17]	2011–2020	Southwestern region	RBPT	199	7	*Brucella* spp.
[18]	NA	Eastern region	Bacterial culture	500	25	*Campylobacter* spp.
[19]	1991–2000	Multiple regions	Microscopic examination	700	62	*Trypanosoma evansi, Dipetalonema evansi, Eperythrozoon* spp.
[20]	2021–2025	Western region	ELISA, PCR	65	13	*Toxoplasma gondii*
[21]	2021–2025	Western region	Blood films, PCR	102	16	*Trypanosomes*
[22]	NA	Central region	Coprological	240	143	*Triehostrongylus* spp. *Haemonehus* spp.*, Triehuris* spp.*, Nematodirus* spp.*, Osrtertagia* spp.*, cestoda: Moniezia expansa, Stilesia* spp.*, Eimeria eameli*
[23]	2001–2010	Eastern region	CIV Test	210	17	*Toxoplasma gondii*
[24]	2011–2020	Eastern region	ELISA	1012	46	BVDV
[25]	2011–2020	Multiple regions	PCR	1369	616	*Anaplasma* and *Piroplasma*
[26]	2011–2020	Western region	Viral microneutralization assays	88	11	Toscana and sandfly fever Sicilian viruses
[27]	NA	Northern region	ELISA, RBPT	308	207	Rotavirus, *Brucella abortus*, *Escherichia coli*, *Salmonella* spp., *Enterococcus* spp.
[28]	NA	Eastern region	WMT, SBS, HCT	600	73	*Trypanosoma evansi*
[29]	2021–2025	Central region	Floatation technique	150	45	*Eimeria rajasthani*
[30]	2011–2020	Multiple regions	ELISA, Vero, Western blot, LIPS	203	150	MERS-CoV
[31]	1991–2000	Central region	Physical examination	860	350	*Cephalopina titilator*
[32]	2001–2010	Central region	PCR	94	16	*Rickettsia, Borrelia*
[33]	2001–2010	Central region	IFAT	412	50	*Neospora caninum, Toxoplasma gondii*
[34]	NA	Central region	IFAT	182	43	*Toxoplasma gondii*
[35]	2011–2020	Central region	PCR	116	1	*Theileria* spp.
[36]	NA	Southern region	Blood films, PCR	679	210	*Trypanosoma evansi*
[37]	NA	Central region	Phenotypic and Genotypic	100	12	*Staphylococcus aureus*
[38]	2011–2020	Central region	ELISA, PCR	171	114	MERS-CoV
[39]	2011–2020	Multiple regions	ELISA	532	117	*Neospora caninum*
[40]	2021–2025	Western region	ELISA	341	172	*Coxiella burnetii*
[41]	2021–2025	Northern region	PCR	8590	46	*Echinococcus granulosus*
[42]	2021–2025	Central region	RBT, CFT, ELISA	274	43	*Brucella* spp.
[43]	NA	Central region	RBT	625	57	*Brucella* spp.
[44]	NA	Western region	Bacterial culture	93	24	*Mycoplasma*
[45]	2011–2020	Southern region	PCR	150	3	*Anaplasma* spp. *Piroplasma* spp.
[46]	NA	Central region	RBPT, STAT, ELISA, CFT	859	102	*Brucella* spp.
[47]	1981–1990	Central region	NA	1680	993	*O. fasciata microfi lariae*
[48]	2011–2020	Northern region	Microscopically	18,525	95	*Echinococcus granulosus*
[49]	NA	Central Province	Bacterial culture	205	118	*Micrococcus* spp., *Staphylococcus aureus*, *Streptococcus* spp., *Corynebacterium* spp.,
[50]	NA	Western region	SWAB	60	4	*Coxiella burnetii*
[51]	NA	Western region	ELISA, PCR	240	192	*Mycobacterium avium*
[52]	2011–2020	Northern region	ELISA, PCR	313	53	*Trypanosoma evansi*
[53]	2011–2020	Multiple regions	ELISA, PCR	1674	1348	MERS-CoV
[54]	2011–2020	Western region	ELISA	1172	270	Hepatitis E Virus
[55]	2011–2020	Eastern region	Molecular techniques	100	26	*Escherichia coli*
[56]	1991–2000	Eastern region	Physical examination	923	480	*Cephalopina titillator*
[57]	1991–2000	Eastern region	Physical examination	103	91	*Sarcocystis*
[58]	NA	Eastern region	ELISA	100	18	*Clostridium perfringens*, *Clostridioides*
[10]	NA	Western region	PCR	44	42	*Anaplasma phagocytophilum*
[59]	1981–1990	Western region	Physical examination	192	64	*Onchocerca fasciata*
[60]	1991–2000	Central region	Blood films	559	131	*Dermatophilus congolensis*, *Microsporum gypseum*
[61]	2011–2020	Eastern region	PCR	67	9	MERS-CoV
[62]	2011–2020	Multiple regions	PCR	180	3	MERS-CoV
[63]	2011–2020	Multiple regions	ELISA	370	11	Peste des petits ruminants virus (PPRV)
[64]	2011–2020	Eastern region	PCR	54	24	MERS-CoV
[65]	2011–2020	Eastern region	PCR	7	5	Retroviruses (5), PPV (0)
[66]	2001–2010	Multiple regions	ELISA	444	32	*Mycobacterium avium* subsp. *Paratuberculosis*
[67]	1981–1990	Central region	Physical examination	2473	1672	*Cephalopina titillator*
[68]	NA	NA	Enzyme immunoassay technique	646	526	*Coxiella burneti, Chlamdophila abortus*
[69]	NA	Multiple regions	Floatation technique	385	154	*Eimeria dromedarii, E. Rajasthani, E. cameli*
[70]	NA	Multiple regions	HAT	227	36	*Toxoplasma gondii*
[71]	2001–2010	Southwestern region	Microscopic examination	140	9	Hydatid cysts
[72]	2011–2020	Western region	PCR	3821	2819	MERS-CoV
[73]	2011–2020	Central region	PCR	698	394	MERS-CoV
[74]	2011–2020	Multiple regions	DFAT	199	158	Rabies virus
[75]	1981–1990	Multiple regions	Microscopic examination	500	208	*Eimeria* spp.
[76]	2011–2020	Eastern region	PCR	96	28	MERS-CoV
[77]	2011–2020	Eastern region	PCR	182	88	*Salmonella* spp., *Campylobacter* spp., *C. burnetii*, *Chlamydia* spp., *Brucella* spp.
[78]	NA	NA	Histopathological	240	31	*Eimeria* spp.
[79]	2011–2020	Multiple regions	PCR	37	15	*Sarcocystis* spp.
[80]	2011–2020	Central region	Microscopic examination	209	75	*Eimeria* spp.
[81]	2021–2025	Central region	PCR	400	171	*Trypanosoma evansi*
[82]	NA	NA	PCR	209	31	*Coxiella burnetii*
[83]	NA	Multiple regions	ELISA	199	68	*Toxoplasma gondii, Neospora caninum*
[84]	1981–1990	Southern region	Physical examination	98	58	*Onchocerca fasciata Railliet & Henry*
[85]	2011–2020	Central region	Digestion technique	80	40	*Sarcocystis* spp.
[86]	2011–2020	Multiple regions	ELISA	121	26	*Clostridium perfringens*
[87]	2011–2020	Central region	Microscopic examination	644	273	*Anaplasma marginale*
[88]	NA	Central region	Higgins and Kock	1000	351	Dermatophilosis, ringworm
[89]	NA	NA	NA	2500	375	*Corynebacterium pseudotuberculosis*
[90]	NA	Central region	RBPT, ABT	2630	342	*Brucella* spp.
[91]	NA	NA	RBPT, ABT	2536	202	*Brucella* spp.
[92]	2011–2020	Multiple regions	PCR	1309	332	MERS-CoV
[93]	2001–2010	Western region	Microscopic examination	541	85	Cystic echinococosis
[94]	NA	Central region	ELISA	376	24	Foot and mouth disease virus

NA: Data not available. BVDV: Bovine viral diarrhoea virus. ELISA: Enzyme linked immune sorbent assay. PCR: Polymerase chain reaction. WMT: Wet Mount Technique. SBS: Stained Blood Smears. HCT: Haematocrit Centrifugation Technique. RBPT: Rose Bengal Plate Test. ABT: Antibody Test/Agglutination-Based Test. HAT: Hemagglutination Test. STAT: Standard Tube Agglutination Test. CFT: Complement Fixation Test. IFAT: Indirect Fluorescent Antibody Test. LIPS: Luciferase Immunoprecipitation System. DFAT: Direct Fluorescent Antibody Test. CIV Test: Camel Immunoglobulin Variable Domain Test.

The pooled prevalence of bacterial infection **(**Fig. 3**)** in camels in Saudi Arabia is calculated from a subset of 36 studies, which accounted for a collective total of 43,795 camels. The reported prevalence of bacterial infections varies considerably across the included studies, ranging from a low of 0.5% [48] to a high of 95.5% [10]. The very high level of heterogeneity in this pooled analysis is evident from an *I*^*2*^ statistic of 99.3% with *P* < 0.001, which portrays high statistical dispersion among all measurements obtained.

**Fig. 3:**
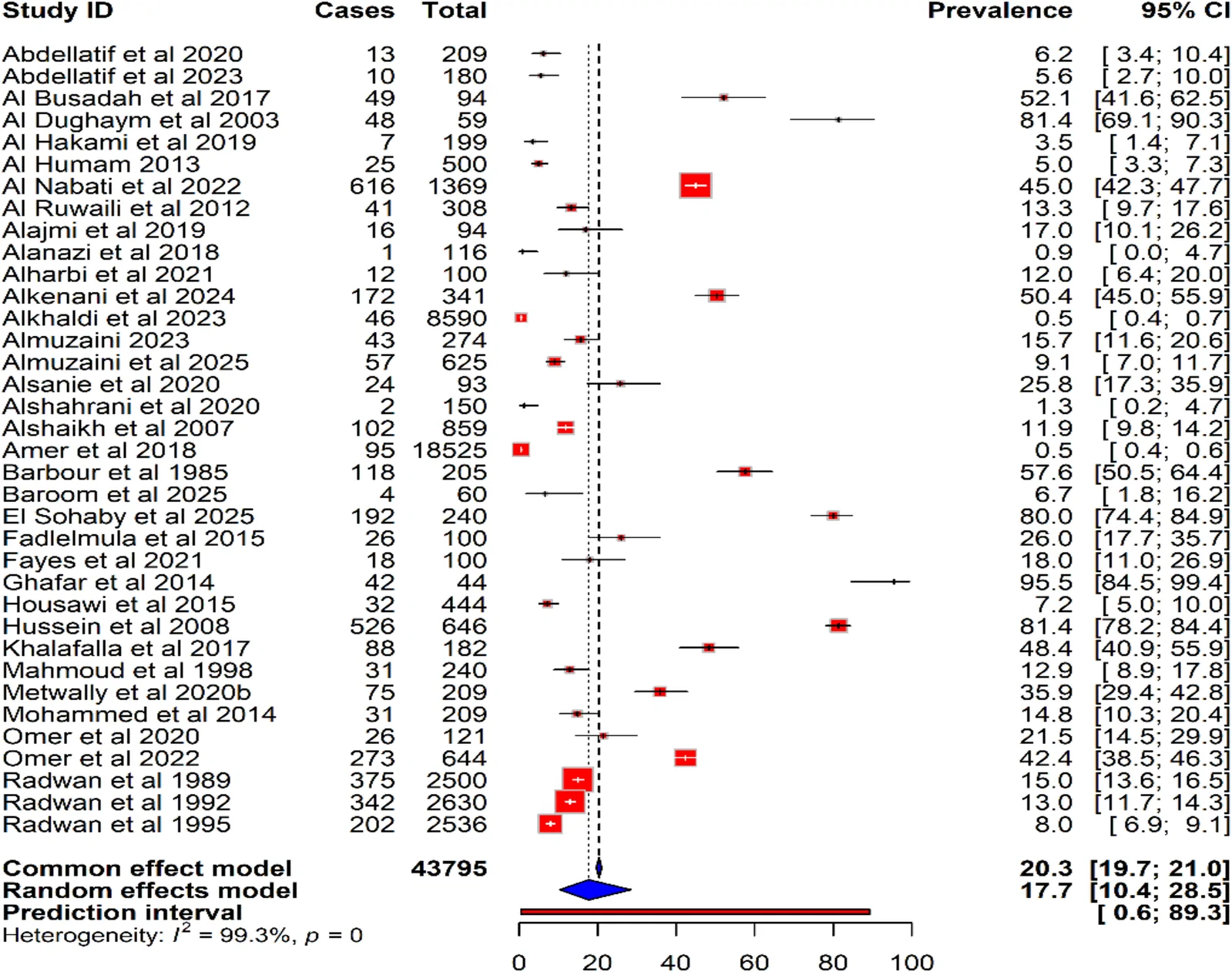
Forest plot of the pooled prevalence of bacterial infection rates among camels in Saudi Arabia. The individual study estimates, along with their 95% CI and raw data (cases/total), are displayed.

A forest plot illustrating the pooled prevalence of parasitic infections among camels in Saudi Arabia is shown in Fig. 4, as reported in 31 individual studies and a total of 17,834 camels. The prevalence of parasitic infections varies widely across the different studies, ranging from a low of 0% [25] to a high of 88.3% [57]. The high *I*^*2*^ statistic of 99.0% (*P* < 0.001) reflects a high level of statistical heterogeneity among the study results. This implies that a major part of variability among individual study outcomes is attributable to factors other than chance and can thus be attributed to varying ecological factors affecting parasitic diseases in camels in Saudi Arabia.

**Fig. 4:**
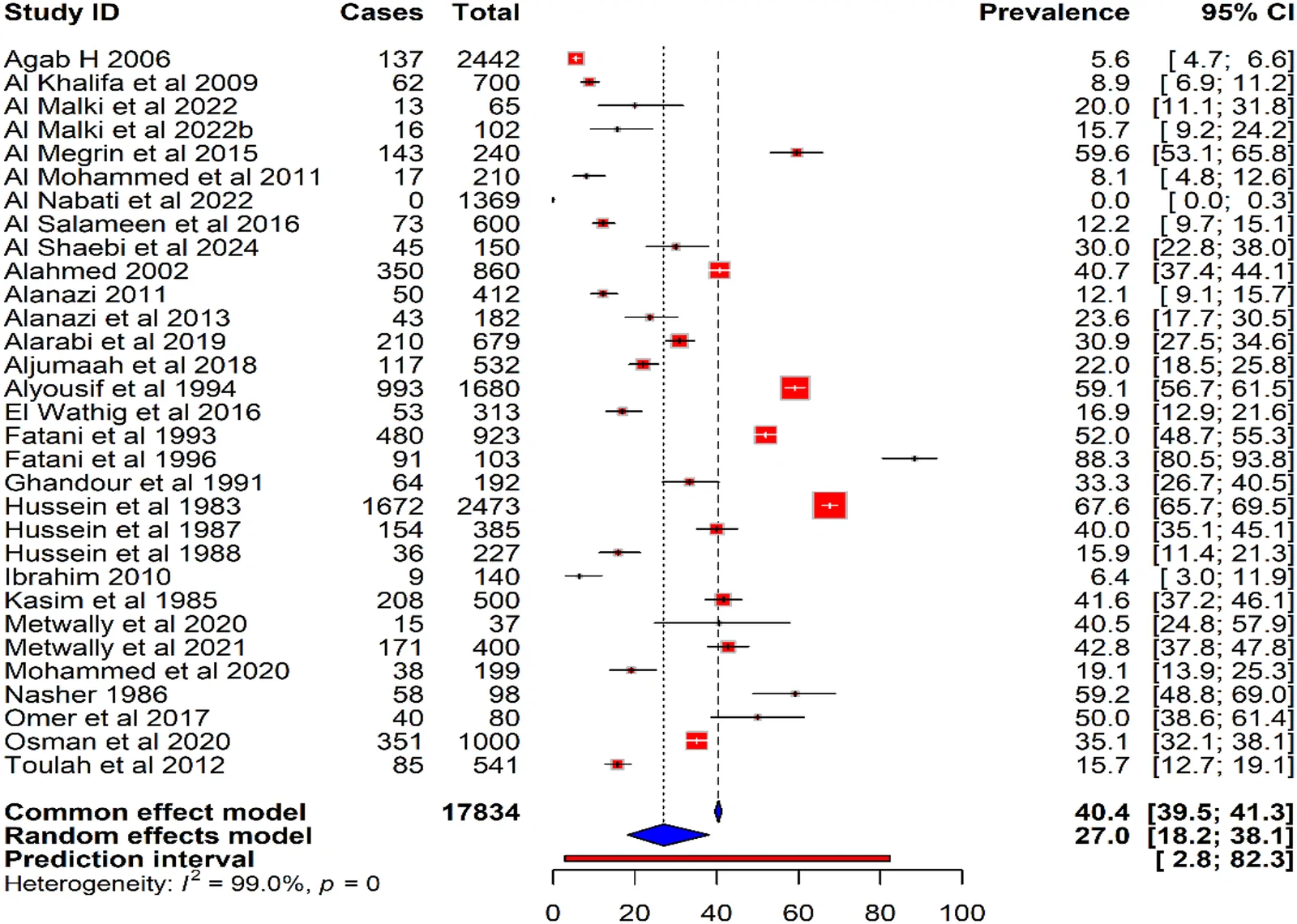
Forest plot of the pooled prevalence of parasitic infections among camels in Saudi Arabia. The plot lists each study with its corresponding prevalence estimate (proportion) and 95% CI.

A forest plot showing the pooled prevalence of viral infection in camels in Saudi Arabia is shown in Fig. 5, obtained from 24 individual studies encompassing a total sample of 21,361 camels. The prevalence of viral infection shows wide variation among studies, with a wide range of prevalence being recorded from 0.2% [9] to 88.0% [95]. The extremely high *I*^*2*^ value of 99.52% (*P* < 0.001) confirms the presence of profound statistical heterogeneity among the study results. This indicates that the variation in prevalence estimates between studies is almost certainly due to real differences in factors beyond chance, such as the circulation of different viruses, outbreak statuses, and varied study methodologies.

**Fig. 5:**
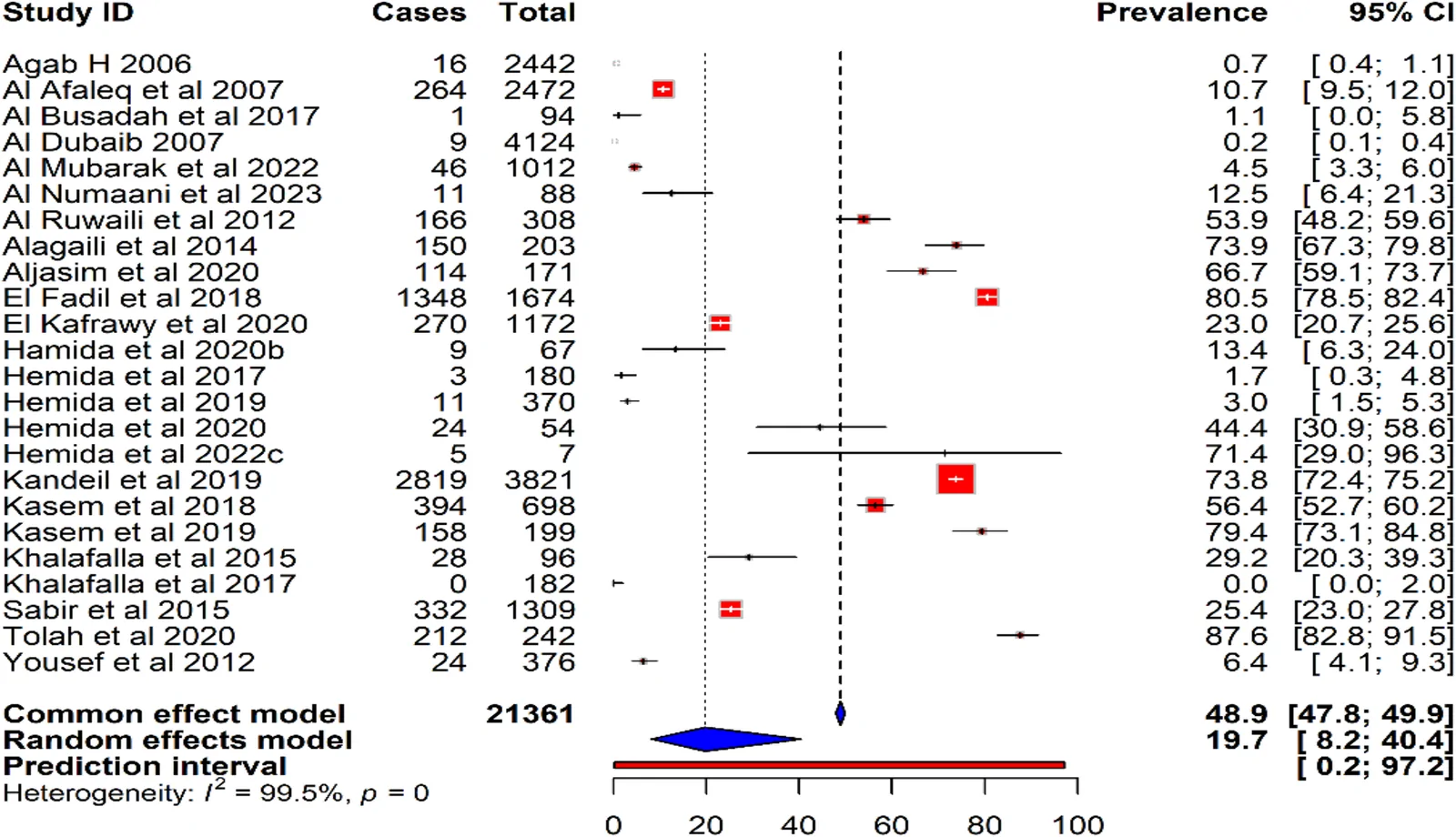
Forest plot of the pooled prevalence of viral infection rates among camels in Saudi Arabia. It gives a summary of individual studies placed along with their respective prevalence estimate (proportion) and 95% CI and shows individual study data used in making this plot (no. of infected camels/total no.).

The pooled prevalence of fungal infections among camels in Saudi Arabia is shown in Fig. 6. The individual study estimates show some variation, with prevalence proportions ranging from 6.8% to 23.4%. The high *I*^*2*^ value of 86.5% (*P* = 0.006) indicates substantial statistical heterogeneity among the few available study results.

**Fig. 6:**
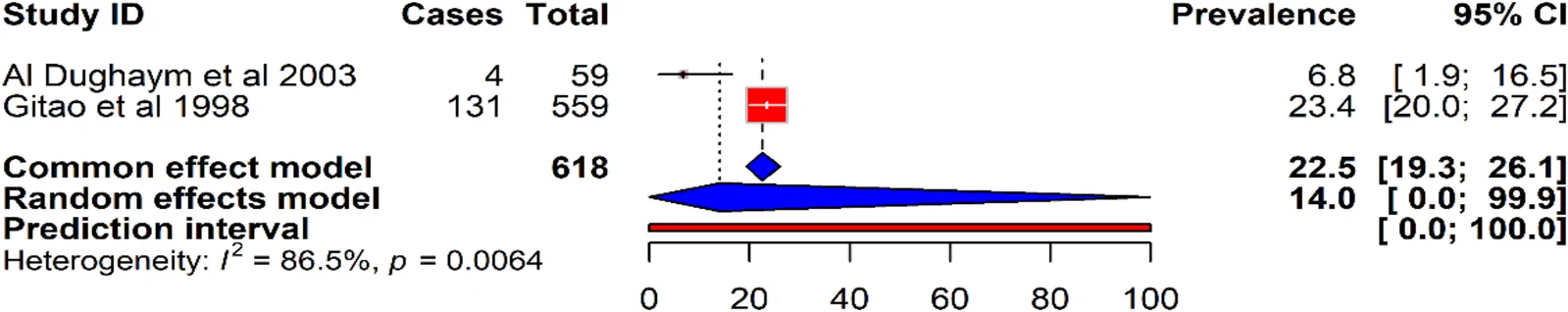
Forest plot of the pooled prevalence of fungal infections among camels in Saudi Arabia. The plot lists the two studies [16], [60] with their corresponding prevalence estimates and 95% CI, along with the raw data (number of infected camels over the total sample size).

The majority (33 studies) were conducted between 2011 and 2020. Geographically, research was most concentrated in the Central region (26 studies), and the most frequently studied pathogens were bacterial (36 studies) and parasitic (31 studies), with fungi being the least investigated (2 studies). The details are represented in Table 2.

**Table 2:** Prevalence of infections among camels in Saudi Arabia according to the different subgroups.

Subgroup	Number of studies	Prevalence (%)	95% CI	Heterogeneity test	
				*I*^*2*^ (%)	*p*-value
2021–2025	8	15.0	0.04–0.42	99.7	<0.001
2011–2020	33	26.0	0.15–0.40	99.5	<0.001
2001–2010	9	10.0	0.03–0.31	97.9	<0.001
1991–2000	5	41.0	0.09–0.84	98.9	<0.001
1981–1990	5	52.0	0.35–0.69	97.8	<0.001
Overall	60	25.8	0.19–0.33	99.3	<0.001

**Region**					
Northern	6	6.0	0.01–0.39	99.7	<0.001
Central	26	24.0	0.15–0.38	99.2	<0.001
Eastern	13	40.0	0.20–0.65	99.2	<0.001
Southwestern	2	5.0	0.00–0.74	33.5	0.220
Western	13	23.9	0.23–0.59	99.2	<0.001
Southern	3	20.0	0.00–0.98	99.6	<0.001
Multiple regions	17	28.0	0.15–0.47	99.1	<0.001
Overall	80	32.7	0.30–0.34	99.81	<0.001

**Organism type**					
Bacteria	36	17.6	0.10–0.28	99.3	<0.001
Viruses	24	19.7	0.08–0.40	99.52	<0.001
Parasites	31	26.9	0.18–0.38	98.97	<0.001
Fungi	2	14.0	0.03–0. 99	86.5	<0.001

The quality assessment of retrieved reports showed that a very high percentage of the assessed studies (between 88% and 100%) were rated favourably (“Yes”) for essential quality metrics. The high “Yes” percentages indicate that the vast majority of studies included in the analysis were deemed to be of high methodological quality according to the JBI standards (Fig. 7).

**Fig. 7:**
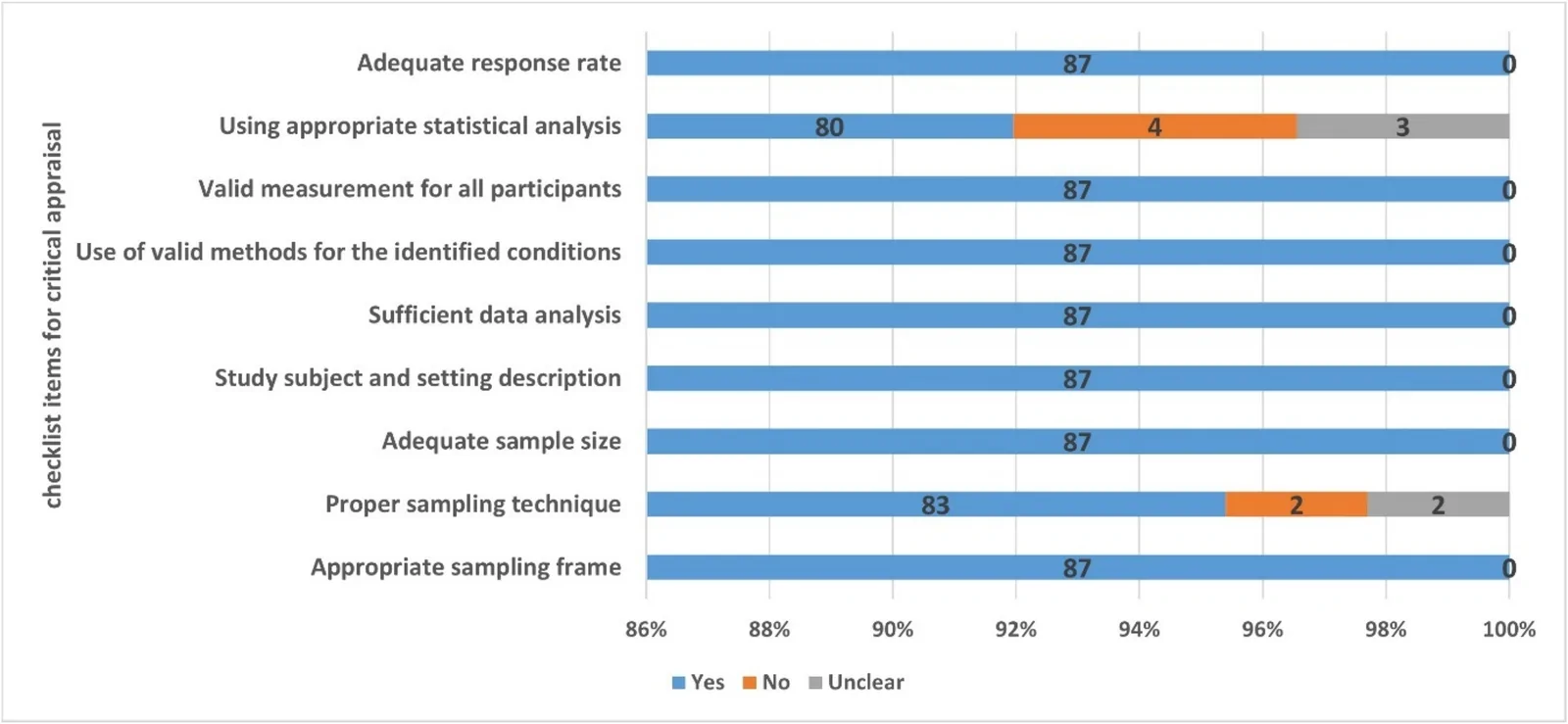
Quality assessment scale for reports included in the study, using the JBI scale.

Egger's regression test indicated significant funnel plot asymmetry (z = −2.15, *p* = 0.032), suggesting the presence of potential publication bias or small-study effects. The funnel plot visually displays the relationship between the logit event rate (effect size) of individual studies and their standard error (a measure of precision). The negative regression coefficient (b = −0.653; 95% CI: −1.206 to −0.100) indicates that studies with larger standard errors (typically smaller studies) tended to report higher effect sizes, contributing to asymmetry in the funnel plot. The limit estimate as the standard error approaches zero remained statistically significant, further supporting the presence of bias, in which smaller studies with a set of results, such as being non-significant or with a lower estimate of prevalence, have a possibility of being underrepresented in a given analysis. Therefore, such a bias may contribute to an exaggeration of a pooled estimate in a meta-analysis, as shown in Fig. 8A.

**Fig. 8:**
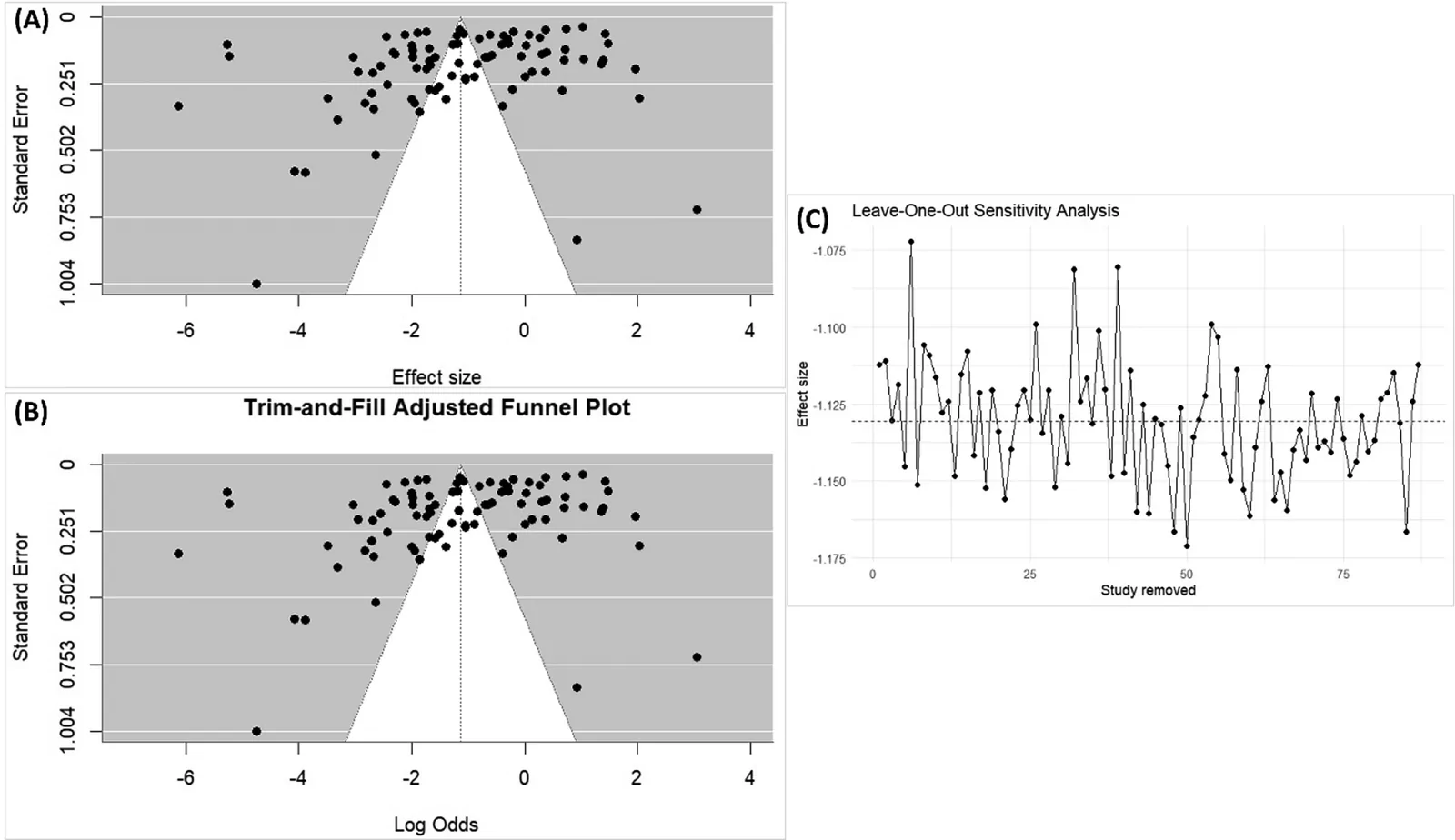
**(A)** Funnel plot showing evidence of publication bias among retrieved reports, supported by Egger's regression test (*p* = 0.032). **(B)** Trim-and-fill analysis for publication bias adjustment. **(C)** Leave-one-out sensitivity analysis showing the influence of individual studies on pooled estimates.

The trim-and-fill analysis did not identify any missing studies (number of imputed studies = 0), indicating that no adjustment to the pooled estimate was required **(**Fig. 8B**)**. The bias-adjusted pooled estimate (logit scale: −1.13; 95% CI: −1.48 to −0.78) remained statistically significant and was comparable to the original model. These findings suggest that, despite the presence of funnel plot asymmetry indicated by Egger's test, the overall pooled estimate was relatively robust to potential publication bias. Additionally, leave-one-out sensitivity analysis demonstrated that the pooled estimate remained stable, with effect sizes ranging narrowly from −1.07 to −1.17 **(**Fig. 8C**)**. No single study exerted a disproportionate influence on the overall estimate. Additionally, heterogeneity remained consistently high across all iterations (I^2^ = 99.5%), indicating that the observed variability was not driven by any individual study.

## 4 Discussion

This study provides a comprehensive synthesis of infectious disease prevalence among camel populations in Saudi Arabia. The findings indicate a substantial overall burden of infection, with marked variability across pathogens and geographic regions. Parasitic infections demonstrated comparatively higher prevalence, while viral and bacterial pathogens of zoonotic importance were also consistently reported. The analysis further revealed significant heterogeneity among studies, reflecting differences in study design, diagnostic approaches, and regional epidemiological patterns. Additionally, evidence of publication bias was observed, suggesting that the pooled estimates should be interpreted with caution. Collectively, these results highlight the complexity of infectious disease dynamics in camels and underscore the need for targeted surveillance and control strategies.

The spread of infectious diseases from domestic and human-associated animals to wildlife is becoming an important global issue due to its economic and environmental consequences [96]. In the Arabian Peninsula, including Saudi Arabia, there has been an increase in reported viral, bacterial, and parasitic diseases that have come into light due to current studies. Many infectious diseases, like hemorrhagic septicemia, tuberculosis, enterotoxemia, chlamydiosis, Q-fever, brucellosis, PPR, theileriosis, CCPP, FMD, and toxoplasmosis have been documented by various analytical methods in Saudi Arabia [97]. The presence of these pathogens in camel populations has important implications for human health, particularly among high-risk occupational groups such as herders, abattoir workers, veterinarians, and individuals involved in camel trade and husbandry. The observed prevalence of these pathogens highlights the need for strengthened surveillance, routine screening of camel herds, and targeted public health interventions, including occupational safety measures, awareness programs, and promotion of safe handling and consumption practices.

The pooled prevalence of infectious diseases among camel populations in Saudi Arabia was estimated at 24% (95% CI: 0.18–0.32), indicating a substantial burden of infection. This estimate is comparable to, and in some cases higher than, prevalence rates reported in other livestock species and regions, where infectious disease burdens often range between 10 and 30% depending on pathogen type and production systems [4], [8]. The relatively elevated prevalence observed in camels may reflect unique ecological and management factors, including extensive grazing systems, frequent animal movement, and close contact with humans and other livestock species. The presence of zoonotic pathogens within this burden amplifies the public health relevance, particularly in settings with close human-camel interactions. Within the One Health framework, these findings highlight the interconnected risks between animal health, human health, and environmental factors, underscoring the need for integrated surveillance and control strategies to mitigate both economic losses and zoonotic transmission.

A notable finding of this analysis is the predominance of parasitic infections compared with bacterial and viral pathogens. This pattern may be attributed to several epidemiological and management-related factors. Parasites, particularly protozoa and hemoparasites, often exhibit strong environmental persistence and complex life cycles that facilitate sustained transmission under extensive husbandry systems [96]. In Saudi Arabia, camel production commonly involves open grazing, herd mobility, and shared water sources, all of which increase exposure to contaminated environments and vectors [3], [54]. In contrast, many bacterial and viral infections are more episodic and may be influenced by outbreak dynamics, vaccination practices, or targeted control interventions [98]. The high prevalence of parasites such as *Eimeria* spp. and *Trypanosoma* spp. further underscores this trend, as these pathogens are well-adapted to arid and semi-arid environments and can persist through environmental contamination or vector-mediated transmission. Within a One Health context, the widespread nature of parasitic infections not only impacts camel health and productivity but also highlights gaps in routine veterinary management and surveillance systems that require targeted intervention.

A high degree of heterogeneity was observed across the included studies (*I*^2^ > 90%), which is not unexpected in prevalence meta-analyses of infectious diseases. This variability can be attributed to several factors, including differences in geographic regions within Saudi Arabia, variations in camel populations, and the diversity of pathogens investigated [53], [62]. Additionally, methodological differences such as study design, sample size, and the use of different diagnostic techniques likely contributed to the observed heterogeneity. Temporal variation in study periods and outbreak-related sampling may have further influenced prevalence estimates. While a random-effects model was applied to account for this variability, the pooled estimates should be interpreted with caution.

Evidence of publication bias was observed, which may reflect the preferential publication of studies reporting higher or statistically significant prevalence estimates. Smaller studies appear to report higher prevalence estimates, which may lead to an overestimation of the pooled prevalence. This bias can lead to an overestimation of the true burden of infectious diseases in camel populations. Although Egger's test indicated significant funnel plot asymmetry, the trim-and-fill method did not impute any missing studies, suggesting that the asymmetry may not be primarily driven by publication bias. Instead, the observed asymmetry is likely attributable to substantial between-study heterogeneity (I^2^ = 99.6%), differences in study design, diagnostic methods, or population characteristics. The leave-one-out analysis confirmed the robustness of the pooled estimate, as exclusion of any single study did not materially alter the results. Therefore, while the possibility of small-study effects cannot be excluded, the pooled prevalence estimate appears relatively stable after bias adjustment.

Substantial regional variation in prevalence was observed, with markedly higher estimates in the Eastern region (40%) compared to the Southwestern region (5%). This disparity likely reflects a combination of epidemiological and methodological factors. The Eastern region is characterized by higher camel population density, intensive farming practices, and active animal movement and importation pathways, which may facilitate pathogen transmission and introduction [25]. In contrast, the lower prevalence reported in the Southwestern region may be influenced by smaller herd sizes, differing ecological conditions, or limited study coverage [36]. Additionally, regional differences in veterinary infrastructure, surveillance intensity, and research focus may contribute to the observed variation. Similar deviations were noted across other regions, reinforcing the heterogeneity of disease distribution [9], [10]. The lower reported prevalence should not be interpreted as absence of risk; rather, it may reflect underreporting or insufficient data. Therefore, further targeted surveillance in regions with lower reported prevalence is necessary to determine whether these figures represent genuinely lower disease burden or are a consequence of limited study availability.

Temporal analysis revealed notable fluctuations in prevalence across decades (e.g., 52% in 1981–1990, 10% in 2001–2010, and 26% in 2011–2020). These variations may not solely reflect true changes in disease burden but are likely influenced by differences in study design, sample size, and diagnostic methodologies over time. Earlier studies may have relied on less specific diagnostic tools, potentially leading to overestimation, while more recent studies incorporating molecular and standardized serological techniques may provide more accurate estimates [53], [62]. Additionally, shifts in research focus toward specific pathogens or outbreak-driven investigations could contribute to variability in reported prevalence. Improvements in veterinary services, disease control programs, and awareness over time may also play a role in reducing infection rates, although inconsistent implementation across regions may explain the observed resurgence in later years. Similar temporal deviations across datasets further emphasize the need for standardized, longitudinal surveillance systems to accurately monitor trends and distinguish methodological artifacts from true epidemiological changes.

The results further support the adoption of One Health–informed veterinary surveillance models that link animal health data with occupational and food safety monitoring. Targeted preventive interventions, including enhancing biosecurity, reassessing vaccination feasibility, and assessing risks at the herd level, are likely to be more effective than blanket control measures in reducing pathogen circulation within camel populations and mitigating zoonotic spillover risk. The observed heterogeneity reflects genuine variation in infection pressure, surveillance intensity, and pathogen ecology across regions and time periods.

### 4.1 Study limitation

This study represents the most comprehensive synthesis to date of infectious diseases affecting dromedary camels in Saudi Arabia, integrating data across four pathogen domains and four decades. The large cumulative sample size strengthens the robustness of prevalence estimates. A key limitation of this study is the substantial variability in diagnostic methods across included studies, including serology, molecular assays, culture, and clinical examination, which differ in their sensitivity, specificity, and ability to distinguish between exposure and active infection. Another potential limitation of this study was that nine relevant articles could not be accessed despite efforts to retrieve full texts. The exclusion of these studies may have introduced selection bias and could potentially influence the pooled estimates. Additionally, the detection of publication bias further suggests incomplete reporting of low-prevalence findings. These limitations highlight the need for standardized surveillance protocols and longitudinal One Health studies to more accurately quantify zoonotic risk.

## 5 Conclusion

This systematic review and meta-analysis demonstrated a considerable and geographically variable burden of infectious diseases among camel populations in Saudi Arabia. The elevated prevalence of parasitic infections, particularly *Eimeria* spp. and *Trypanosoma* spp., highlights the need for targeted, region-specific control strategies, including rotational deworming programs, improved herd management, and enhanced sanitation practices, especially in intensive production systems. In addition, the presence of viral and bacterial pathogens of zoonotic importance underscores the necessity for strengthened biosecurity measures, routine screening, and coordinated surveillance at the human-animal interface. Implementation of integrated One Health approaches linking veterinary and public health sectors will be critical for early detection and prevention of disease transmission.

Considering the evidence of publication bias identified in this study, we recommend the establishment of a national, open-access camel disease surveillance database to ensure comprehensive reporting of epidemiological data, regardless of study outcomes. Such an initiative would improve the accuracy of risk assessments and support evidence-based policy decisions. Future research should prioritize longitudinal and regionally representative studies, particularly in underrepresented areas, and adopt standardized diagnostic approaches, including harmonized molecular and serological methods, to enhance comparability across studies and over time. Collectively, these measures will strengthen veterinary surveillance systems and contribute to more effective control of infectious diseases in camel populations in Saudi Arabia.

The following are the supplementary data related to this article.

Supplementary material The detailed search strategies for each database, including Boolean operators and applied filters, are provided in Supplementary File 1 to ensure transparency and reproducibility.

## CRediT authorship contribution statement

**Ahmad T. Sabri Sabbah:** Software, Methodology, Investigation, Formal analysis, Conceptualization, Writing – original draft. **Mai Abdel Haleem A. Abusalah:** Validation, Software, Resources, Project administration, Data curation, Writing – review & editing. **Alqassem Hamdallah Abuarqoub:** Validation, Software, Resources, Formal analysis, Data curation, Writing – original draft. **Anwar Noor Althubyani:** Software, Resources, Methodology, Formal analysis, Data curation, Writing – original draft. **Fatmah Abdualrzg Albalwi:** Software, Resources, Methodology, Formal analysis, Data curation, Writing – original draft. **Naveed Ahmed:** Visualization, Validation, Supervision, Software, Resources, Project administration, Investigation, Data curation, Conceptualization, Writing – review & editing.

## Declaration of competing interest

Alqassem Hamdallah Abuarqoub is affiliated with Al-Wafi Group for Marketing and Int'l Trade Co. Ltd. The company had no role in the design, conduct, manuscript preparation, or decision to publish this review article. The authors declare that they have no known competing financial interests or personal relationships that could have appeared to influence the work reported in this paper.

## Data Availability

Data will be made available on request.
