# Supplementary material for: Epidemiological patterns of infectious and zoonotic diseases among dromedary camels in Saudi Arabia: a systematic review and meta-analysis of 79,154 camel samples
Source: One Health. 2026 Oct 13;23:101618. doi: 10.1016/j.onehlt.2026.101618 (PMC13691312; doi:10.1016/j.onehlt.2026.101618)
Supplement: Supplementary file 1 [file mmc1.docx]

Search Strategy Document

# Search Strategies by Database:

**Search Strategy for Major Databases:**

1. **PubMed (MEDLINE) (n=154)**

("Camelus dromedarius"[Title/Abstract] OR "dromedary camel"[Title/Abstract] OR "camel"[Title/Abstract])

AND

("infection"[Title/Abstract] OR "infections"[Title/Abstract] OR "infectious diseases"[Title/Abstract] OR "bacterial infection"[Title/Abstract] OR "viral infection"[Title/Abstract] OR "fungal infection"[Title/Abstract] OR "zoonosis"[Title/Abstract] OR "zoonotic diseases"[Title/Abstract])

AND

("Saudi Arabia"[Title/Abstract] OR "Arabian Peninsula"[Title/Abstract])

2. **Scopus (n=354)**

("camel" OR "dromedary camel" OR "Camelus dromedarius")

AND

("infection" OR "infections" OR "infectious diseases" OR "bacterial infection" OR "viral infection" OR "fungal infection" OR "zoonosis" OR "zoonotic disease")

AND

("Saudi Arabia" OR "Arabian Peninsula")

3. **Web of Science (n=235)**

TS=("camel" OR "dromedary camel" OR "Camelus dromedarius")

AND

TS=("infection" OR "infections" OR "infectious diseases" OR "bacterial infection" OR "viral infection" OR "fungal infection" OR "zoonosis" OR "zoonotic disease")

AND

TS=("Saudi Arabia")

1. **Google Scholar Search String (n=14)**

allintitle:("Camel" OR "Dromedary Camel" OR "Camelus dromedarius") AND ("infection" OR "infections" OR "bacterial infection" OR "viral infection" OR "fungal infection" OR "zoonotic disease") AND ("Saudi Arabia" OR "Arabian Peninsula")

2. **ScienceDirect (Elsevier) Search String (n=46)**

("camel" OR "Camelus dromedarius")

AND ("infection" OR "bacterial infection" OR "viral infection" OR "fungal infection" OR "zoonosis" OR "zoonotic disease")

AND ("Saudi Arabia")

3. **Cochrane Library Search String (n=1)**

(“camel” OR "dromedary camel" OR "Camelus dromedarius")

AND (“infection” OR “infections” OR "bacterial infection" OR "viral infection" OR "fungal infection" OR "zoonosis" OR "zoonotic disease")

AND ("Saudi Arabia" OR "Arabian Peninsula")
